# SFOL Pulse: A High Accuracy DME Pulse for Alternative Aircraft Position and Navigation

**DOI:** 10.3390/s17102183

**Published:** 2017-09-22

**Authors:** Euiho Kim, Jiwon Seo

**Affiliations:** 1 Department of Mechanical & System Design Engineering, Hongik University, 94, Wausan-ro, Mapo-gu, Seoul 04066, Korea; 2 School of Integrated Technology, Yonsei University, 85 Songdogwahak-ro, Incheon 21983, Korea; 3 Yonsei Institute of Convergence Technology, Yonsei University, 85 Songdogwahak-ro, Incheon 21983, Korea

**Keywords:** distance measuring equipment, genetic algorithms, APNT, GNSS

## Abstract

In the Federal Aviation Administration’s (FAA) performance based navigation strategy announced in 2016, the FAA stated that it would retain and expand the Distance Measuring Equipment (DME) infrastructure to ensure resilient aircraft navigation capability during the event of a Global Navigation Satellite System (GNSS) outage. However, the main drawback of the DME as a GNSS back up system is that it requires a significant expansion of the current DME ground infrastructure due to its poor distance measuring accuracy over 100 m. The paper introduces a method to improve DME distance measuring accuracy by using a new DME pulse shape. The proposed pulse shape was developed by using Genetic Algorithms and is less susceptible to multipath effects so that the ranging error reduces by 36.0–77.3% when compared to the Gaussian and Smoothed Concave Polygon DME pulses, depending on noise environment.

## 1. Introduction

It is well known that the signals used in Global Navigation Satellites Systems (GNSS) can be easily lost due to radio frequency interference [1,2,3,4,5] or ionospheric anomalies [6,7,8,9]. As modern air traffic control systems like the Next Generation Air Transportation System in the USA are heavily dependent on GNSS [10,11,12] to meet escalating air traffic demands, the vulnerability of GNSS has become a possible threat. For this reason, the Federal Aviation Administration (FAA) has investigated possible Alternative Position, Navigation, and Timing (APNT) systems to maintain safe air traffic control operations during outages of GNSS.

To support APNT, various approaches have been proposed [13,14,15,16,17,18,19]. First, the Mosaic/DME system in Reference [13] provides distance and bearing to an approaching aircraft using a single station that consists of a Distance Measuring Equipment (DME) ground transponder and multiple GPS-like pseudolites. Although 3D positioning is possible with the proposed Mosaic/DME system, its achievable positioning accuracy is very limited due to the poor geometry between pseudolites and the aircraft. Wide Area Multilateration (WAM) using Automatic Dependent Surveillance-Broadcast (ADS-B) signals has also been considered [14,15]. The WAM computes aircraft position by using the time difference of the ADS-B signals received at the ground receivers. The main challenge of the ADS-B based WAM is that the ground and airborne transceivers are not designed for positioning, therefore there are significant hardware and software changes in both the ground transceivers and avionics. An enhanced DME using DME carrier phase measurement as ranging sources has been proposed in [16,17]. This approach can result in a centimeter level of ranging accuracy and requires a significantly more stable oscillator than the one that current DMEs are typically equipped with. More recently, Thiasiriphet et al. proposed modifying future L-band digital aeronautical communication system type 1 (LDACS1) signals as a ranging source for APNT [18,19]. The flight tests in Reference [18] showed that the achievable ranging accuracy was 21 m to 85 m (95% confidence) depending on aircraft altitudes, which may result in a 10–42 m horizontal positioning accuracy with four ground stations.

While these systems can be considered for a long term APNT solution, FAA announced its plan to use DME (DME/N or DME normal) as a short-term back-up solution to GNSS [20]. DME is a conventional pulse ranging system for aircraft that has been used since the 1950s [21], and consists of a ground transponder and an airborne interrogator that measures the slant range between them by exchanging a pair of pulses. When two or more ground transponders are in view of the aircraft, the aircraft horizontal position can be computed by using the slant ranges and the known coordinates of the DME ground transponders, whose positioning technique is referred to as DME/DME. As DME has been used for aircraft navigation since the 1950s, a widespread DME network exists in the Continental United States (CONUS). In addition, the mature technology and operational experience of DME offers a competitive advantage over other APNT alternatives.

Recently, the feasibility of the DME/DME based APNT architecture has been investigated [22,23,24,25] and it was found that the FAA’s desired APNT positioning requirement would hardly be met with today’s DME performance mainly due to its poor ranging accuracy. The reason for the poor ranging accuracy is largely due to the Gaussian pulse waveform typically employed in most DMEs. The ranging accuracy of this waveform satisfies the conventional use of DME, but is not sufficient for APNT. Therefore, an alternative DME/N pulse has been recommended to improve the range accuracy of the DME [23]. Along this line, an alternative DME pulse called the Smoothed Concave Polygon (SCP) pulse was proposed in References [26,27]. This pulse can significantly mitigate the DME multipath effects so that the multipath-induced range error of the SCP pulse is about half of the conventional Gaussian pulse. The SCP pulse was developed by inspecting various asymmetric Gaussian distribution, smoothed trapezoid, and smoothed concave hexagon shapes. Although the SCP pulse significantly exceeds the ranging accuracy of the Gaussian pulse, the design process of the SCP pulse only considers a small search space among possible DME pulses. Therefore, other alternative DME pulses that perform better than the SCP pulse may exist if an advanced optimization technique is used.

This paper presents another promising alternative DME pulse developed by using Genetic Algorithms (GA) that is able to inspect a large search space and has a high chance of finding a global optimum [28,29,30]. The presented pulse—designed using GA—is more robust to multipath effects than the Gaussian and SCP pulses, thus overall DME ranging accuracy can be significantly enhanced. The details of the pulse design elements and the resultant alternative DME pulse from the proposed process are introduced, and the ranging performance of the developed pulse against noise and multipath analyzed.

## 2. Distance Measuring Equipment (DME) Ranging and Pulse Shape Requirements

DME consists of a ground transponder and an airborne interrogator, and measures the slant range between them from the time elapsed when exchanging a pair of pulses. When measuring the time of flight of the pulses, the timing reference is the half amplitude point of the rising edge in the first pulse. When a received pulse is distorted due to noise or multipath, the original half amplitude point of the transmitted pulse shifts, thus leading to potential ranging errors. The level of the half amplitude point shifts is largely dependent on the pulse shape.

The current DME standards specify the pulse shape requirements of rising time, width, falling time, and spectrum power. The DME pulse shape requirements of the ground transponder are listed in Table 1 [31]. The rise time of a pulse is defined as the time elapsed to rise from 10% to 90% of the peak pulse amplitude in the leading edge. The fall time is the time elapsed to fall from 90% to 10% of the peak pulse amplitude in the trailing edge. The pulse duration, or width, is the time between the half amplitude points in the leading and trailing edges.

The spectrum power requirement is based on the effective radiated power (ERP) within a 0.5 MHz band that is centered on the frequencies ±0.8 MHz and ±2.0 MHz away from the nominal DME channel frequency. The ERP on frequencies ±0.8 MHz away from the nominal channel frequency should not exceed 200 mW (23 dBm). Furthermore, the ERP on frequencies ±2 MHz away from the nominal channel frequency should not exceed 2 mW (3 dBm). In addition, the ERP contained within any 0.5 MHz band should decrease monotonically as the band center frequency moves away from the nominal channel frequency.

The ERP is defined as the product of the power supplied to the antenna and the antenna gain relative to a half wave dipole in a given direction [32]. The power supplied to the antenna in the requirement is the average power in a 0.5 MHz frequency band centered on either ±0.8 MHz or ±2 MHz away from the center frequency of a DME channel. Then, the ERP, *P_ERP_*, can be formulated as follows

(1) $$P_{ERP}=P_{Avg}+G_{Ant}+EIRP_{Conv}+L_{Cable}+D\;\mathrm{in}\;\mathrm{dB}\;$$

where *P_Avg_* is the average transmission power; *G_Ant_* is antenna gain; *EIRP_Conv_* is the conversion factor between ERP and Effective Radiated Isotropic Power (EIRP); *L_Cable_* is the cable loss from the DME transmitter to an antenna; and *D* is the duty cycle factor that is the ratio of average power to peak power. Equation (1) was used to compute the ERP of a new DME pulse described later in this paper.

**Figure 1:**
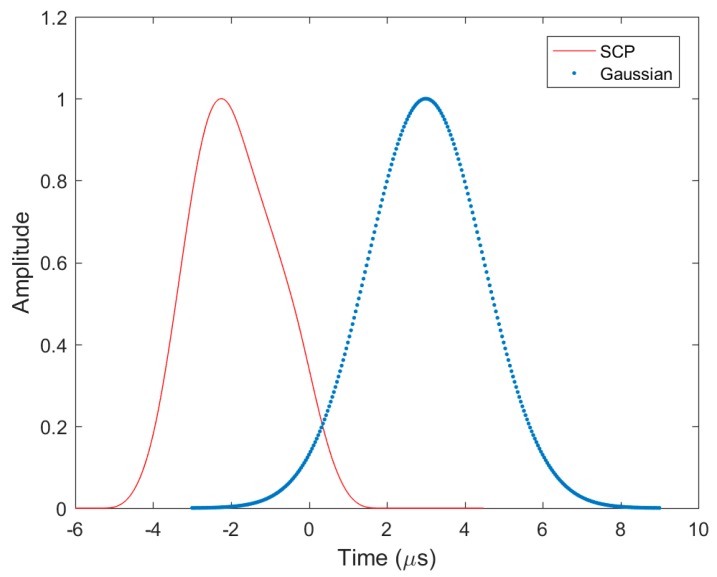
Gaussian and Smoothed Concave Polygon DME pulses.

Figure 1 shows a traditional Gaussian DME pulse and a recently developed smoothed concave polygon (SCP) pulse [26]. Gaussian pulse has been traditionally used in practice, and the SCP pulse was developed to overcome the poor ranging performance of the Gaussian pulse. These two pulses satisfy the above pulse shape and spectrum requirements.

## 3. Baseline Approach Using Genetic Algorithms for a DME Pulse Design

In optimization problems, if the derivative information from a cost function is available, a calculus-based optimization technique should be used. In other cases (including the pulse shaping problem), Genetic Algorithms (GA) have a great chance of solving challenging optimization problems [29]. There are several key elements in GA that are needed to tackle a given problem such as chromosomes, genes, population, fitness function, offspring, generation, and mutation. These elements were tailored for a pulse shaping problem that will be discussed in this section.

GA have been applied to pulse design problems in various fields. Baumert et al. designed a femtosecond laser pulse shape by using a feedback-based evolutionary algorithm [33,34]. Turhan-Sayan et al. developed an optimal pulse shape that could enable natural resonance based target identification [35,36]. Yong et al. used GA to optimize the multidimensional spatial selective Radio Frequency (RF) pulse to reduce the passband and stopband errors of an excitation profile, which would enhance image quality in Magnetic Resonance Imaging (MRI) equipment [37].

### 3.1. Chromosome Definition

**Figure 2:**
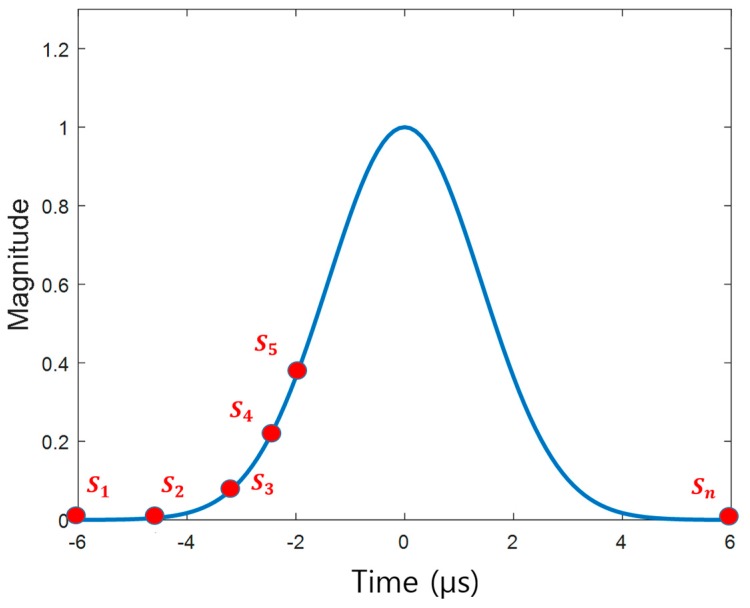
Pulse sampling points can be modeled as chromosomes.

The first important step in a GA problem is to determine the definition of a chromosome. For the pulse design problem, References [33,34,35,36] used an indirect approach where a chromosome evolution was performed on the characteristic of the equipment generating a pulse, for example a spatial light modulator. On the other hand, Reference [37] used a direct approach where a chromosome represented the RF pulse amplitudes. In this DME pulse design problem, it was natural to represent pulse amplitude at designated times as a chromosome where

(2) $$chromosome=[s_{1},s_{2},s_{3},\cdots ,s_{n}]$$

where *n* is the length of the chromosome, and *s* is a gene. Therefore, the genes were pulse samples with value ranges from 0 to 1 as the peak amplitude was normalized to 1 as shown in Figure 2. For efficient computation time, the length of the chromosome was kept minimal between 60 and 80, and a piecewise cubic spline function was used to interpolate the intervals between the samples and generate a smooth pulse shape.

### 3.2. Initial Population

In GA, an evolution process is implemented by a group of chromosomes called population as described below

(3) $$Population=\left[\begin{array}{c} chromosome_{1}\\ chromosome_{2}\\ \vdots \\ chromosome_{m} \end{array}\right]$$

Within a population, superior chromosomes are selected as parents and they mate with each other to result in a following generation. Next, the superiority of chromosomes is determined by a fitness function that is discussed in the next subsection. Therefore, it is necessary to generate an initial population at the beginning of the GA process. In this work, an initial population was generated from the random numbers uniformly drawn from the designated ranges for each gene. This range was called an initial population range and helped the overall GA optimization process find a valid pulse shape that met the DME pulse shape requirements. The initial population range was given as a 2 × n matrix

(4) $$Initial\;Population\;Range=\left[\begin{array}{c} p\\ q \end{array}\right]=\left[\begin{array}{cccc} p_{1} & p_{2} & \cdots & p_{n}\\ q_{1} & q_{2} & \cdots & q_{n} \end{array}\right]$$

where the vector *p* followed an asymmetric Gaussian distribution as follows

(5) $$p_{i}\left(t_{i},t_{o},\sigma_{R},\sigma_{F}\right)=\{\begin{array}{c} e^{-\frac{{\left(t_{i}-t_{o}\right)}^{2}}{2\sigma_{R}^{2}}}\;if\;t_{i}\leq t_{0},\\ e^{-\frac{{\left(t_{i}-t_{o}\right)}^{2}}{2\sigma_{F}^{2}}}\;otherwise \end{array}$$

where *t*_i_ is the sampling time; $t_{o}$ is the mean; $\sigma_{R}$ is the standard deviation in the rising edge; and $\sigma_{F}$ is the standard deviation in the falling edge, respectively. Then, vector q is determined from

(6) q=ρ·p

where ρ is a pulse magnitude scaling facor set between 0 and 1.

**Figure 3:**
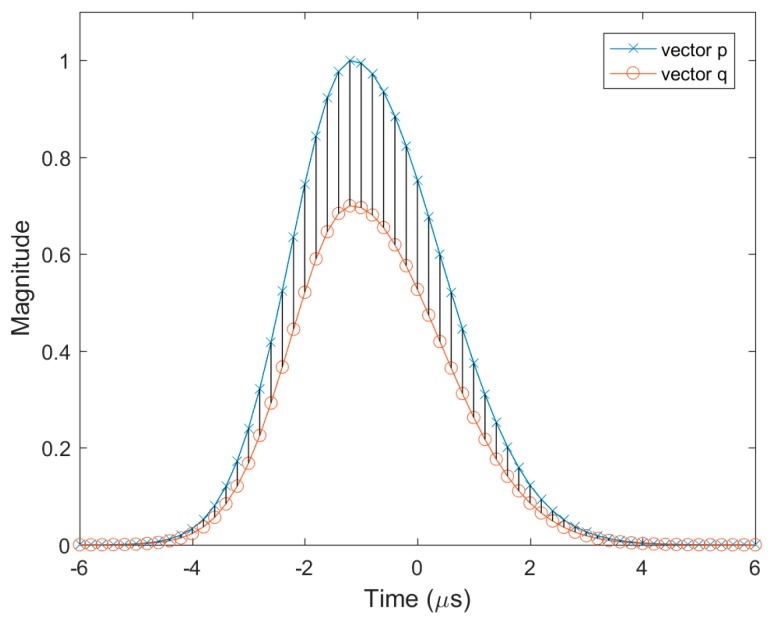
The initial population range consisting of vectors *p* and *q*. The genes in the initial population can have values in the shaded region.

In addition, $\sigma_{R}$ is smaller than $\sigma_{F}$, and this drives the pulses in the initial population to have a fast rise time that would make pulses more resistant to noise and multipath effects [26]. Figure 3 shows an example of an initial population range where $\sigma_{R}=1.05\;\mu s$, $\sigma_{F}=5.02\;\mu s$, $t_{o}=-1.2\;\mu s$, and ρ=0.7. Note that the given initial population range only governed the chromosomes in the initial population. The chromosomes in the following generations were not limited by the initial population ranges.

### 3.3. Fitness Function

The chromosomes in a population were evaluated with respect to a cost or fitness function. During the evaluation, the fitness function assigned a cost to each chromosome. In this DME pulse design problem, there were two factors that determined cost: compliance with the DME pulse shape requirements, and resultant range accuracy under multipath. If a chromosome did not comply with any of the pulse shape requirements, a large penalty was assigned to the chromosome. For chromosomes with a valid DME pulse shape, its ranging accuracy under multipath was assigned as a cost. The reason for using the ranging accuracy under multipath is that multipath is the largest range error source in the DME [23,24] and multipath-induced range errors are primarily determined by pulse shape.

To assess the multipath-induced range error of a candidate pulse in the fitness function, a DME multipath in a baseband, m(t), was modeled as follows

**Figure 4:**
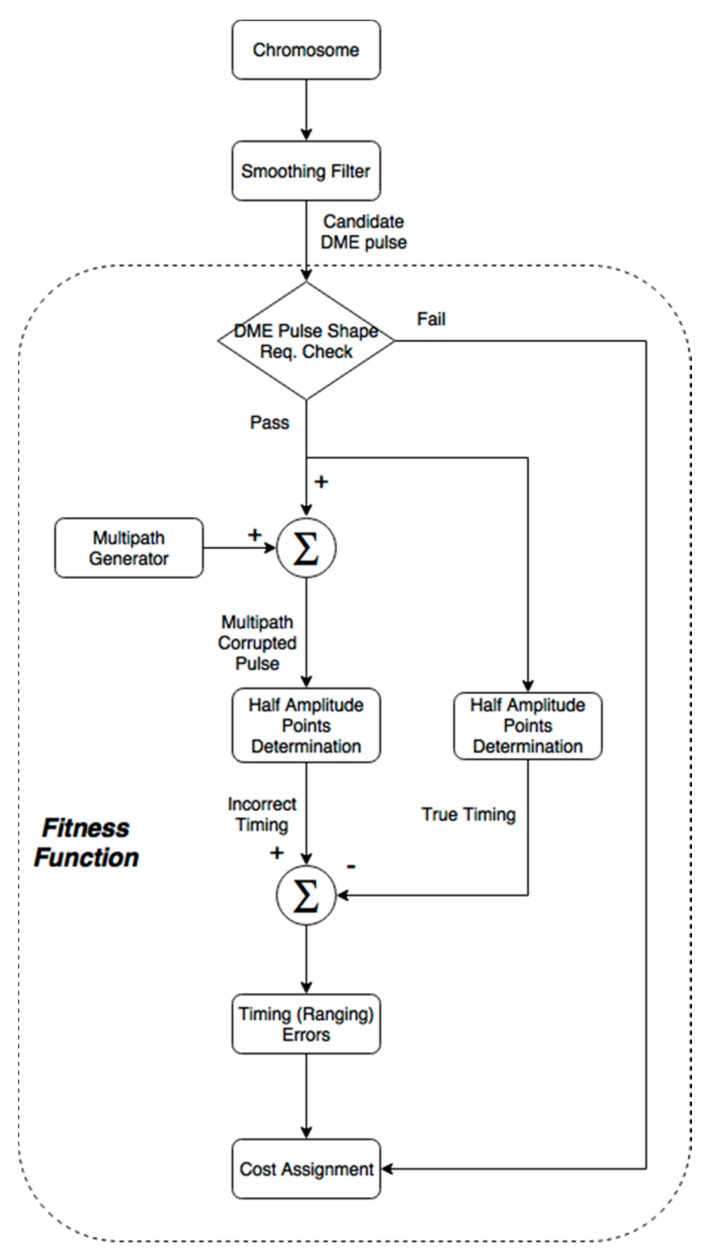
Fitness function that assess the ranging accuracy of a DME pulse chromosome with respect to multipath.

(7) m(t,ϕ,δ)=αcos(ϕ)x(t−δ)

where *t* is the sampling time, and α is the peak amplitude ratio of the multipath to the direct. ϕ is the relative phase difference between the direct to the multipath, which ranged from 0 to 2π. δ is the time delay of the multipath with respect to the direct pulse and ranged from 0 μs to 6 μs as a multipath beyond 6 μs barely distorts the rising edge of the direct pulse. x is the candidate pulse shape from a chromosome. Next, a cost due to multipath effects was computed from

(8) $$C=\sqrt{\frac{1}{I\cdot J}\sum_{i=1}^{I}\sum_{j=1}^{J}{\left(\text{Η}\left(x,x+m\left(\frac{\pi i}{I},\frac{\delta_{\max}j}{J}\right)\right)\right)}^{2}}$$

where δ_max_ is the maximum delay that can impact the half amplitude point, which is around 6 μs. In Equation (8), Η(χ,ψ) is a function that determines the range errors based on the timing difference of the half amplitude point in the rising edge between the direct, χ, and overlapped, ψ, pulses. In practice, Η(χ,ψ) numerically finds the timing error by comparing the half amplitude points in the rising edge of the direct and overlapped pulses. Furthermore, the ranges of ϕ and δ are discretized to small quantities and there are a total I times J combinations of multipath. Note that fitness is the inverse of cost, and Figure 4 depicts the overall fitness function process.

### 3.4. Natural Selection, Paring, Mating, and Mutation

The fitness assigned to each chromosome in the fitness function was used to select parents that will mate to produce the next generation. In this work, the selection criterion for parents was based on a roulette wheel weighting where the fitness was translated to the area of the roulette [29]. The higher cost a chromosome, the smaller the area assigned. Then, a roulette spin was simulated where a chromosome with a large area was selected as parents with a higher probability. Typically, forty percent of the population were selected as parents, and the rest discarded. An exception was that the elite, the chromosome with the minimum cost, was guaranteed to survive to the next generation as a parent.

The survived parents in the previous step mated and produced an offspring in the next generation. Therefore, forty percent of the new generation was made up of parents and the remaining sixty percent was offspring. In this work, a mother and a father were randomly chosen from a uniform distribution regardless of their cost ranks assigned from the fitness function. For the mating process, a heuristic crossover of the parents is commonly used in the continuous GA algorithms [29,30] and was also applied in this paper. Let us assume that the survived parent chromosomes are

(9) $$\begin{array}{c} father=[f_{1},f_{2},\cdots ,f_{n}]\\ mother=[m_{1},m_{2},\cdots ,m_{n}]. \end{array}$$

Then, the heuristic crossover produces an offspring from the two parents as follows

(10) $$s_{i}=f_{i}+\tau \gamma (f_{i}-m_{i})$$

where

(11) $$offspring=[s_{1},s_{2},\cdots ,s_{n}].$$

In Equation (10), τ is a random number from a uniform distribution having a range from 0 to 1. γ is a variation ratio and its typical value ranges from 2 to 4.

The GA prevents variables from quickly converging to a local region of cost surfaces through a process called mutation. Mutation explores other parts of the cost surfaces by randomly changing the values of some genes. The mutation process is controlled by a mutation rate, υ, that is typically between 0.2 and 0.3. Given the mutation rate, the GA randomly (uniform distribution) selects k number of genes in a chromosome from the following equation

(12) k=⌊υ⋅n⌋.

Next, the selected *k* genes are assigned a new value generated from a uniform distribution. In this case, the range of the uniform distribution was determined from the minimum and the maximum values for the selected genes in the populations of the current generation.

## 4. Results

### 4.1. Optimal DME Pulse Developed from the Genetic Algorithms Process

To implement the above GA algorithms, the MATLAB Genetic Algorithms Toolbox was used as a solver. A chromosome length of 60 was chosen as discussed in Section 3.1. G*_Ant_* was set to 9 dB, which is an antenna gain typically employed in a high power DME to provide a DME service within 200 nautical miles. The duty cycle, D, was set to 16.21 dB after considering the maximum transmission rate of 4800 pulse pairs per second in modern DMEs. EIRP_Conv_ was set to −2.15 dBi as a fixed conversion factor between ERP and EIRP. The cable loss, L_Cable_, was set to −2.6 dB by assuming a 3 m coaxial transmission line and 0.86 dB/m loss at 1 GHz. These parameters were used to compute the ERP during the pulse transmission in Equation (1).

**Figure 5:**
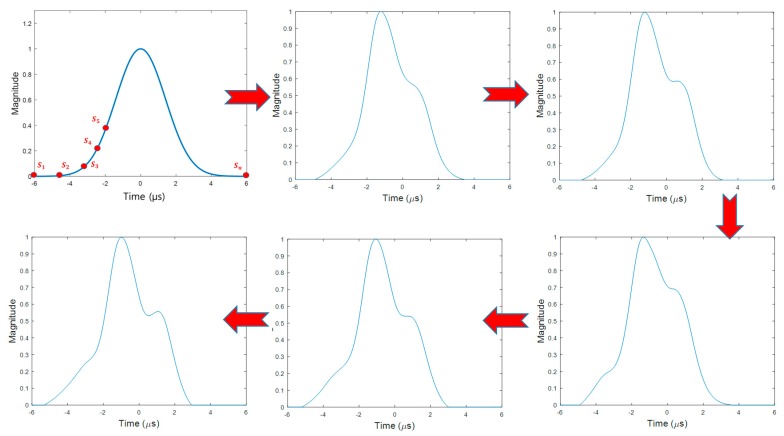
DME pulses developed during the evolution process.

**Figure 6:**
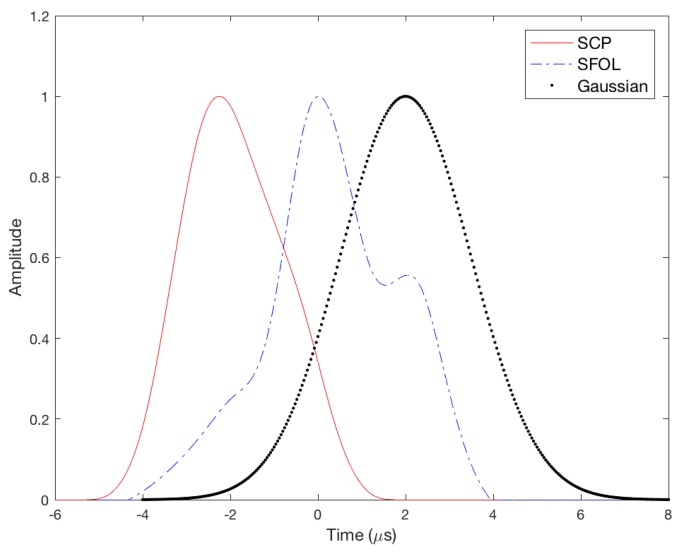
Comparison of the Smoothed Concave Polygon (SCP), Stretched-Front-Leg (SFOL), and Gaussian DME pulses.

As discussed in Section 3, the proposed GA process started with a random initial population and the fitness in each chromosome was evaluated in the fitness function. Chromosomes that survived their fitness evaluation produced the next generation by implementing the paring, mating, and mutation algorithms. This process iterated as long as a better chromosome was found during 50 consecutive generations. Otherwise, the process terminated. In general, the GA process does not always yield exactly the same results in every implementation due to the randomness allowed in the GA algorithms. However, the pulse evolution overall follows a similar pattern as shown in Figure 5, and Figure 6 shows the converged optimal pulse called the Stretched-FrOnt-Leg (SFOL) pulse with the previous two DME pulses. The SFOL pulse has a rise time of 2.8 μs, a width of 3.4 μs, and a falling time of 3.0 μs, which satisfies the pulse shape requirements in the current DME ground transponder specifications. The overall shape of the SFOL pulse is very different from the other two pulses. Most notably, its rising edge starts relatively slowly in the beginning and rapidly goes up toward the peak amplitude. Additionally, there is a big hump in the falling edge. The unique shape of the rising edge turned out to be very effective in reducing multipath effects (as will be discussed later in this subsection). The role of the hump in the falling edge is mainly to have the width and falling time meet the DME standards.

**Figure 7:**
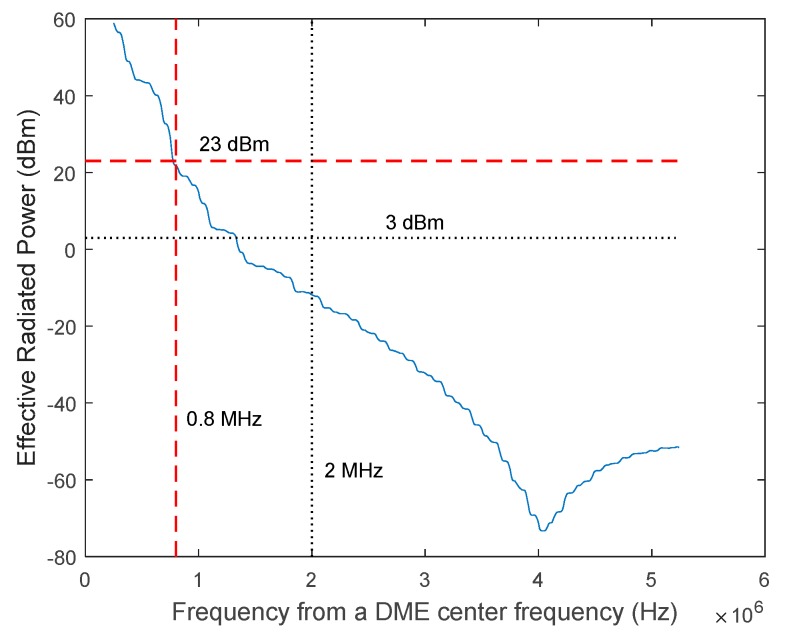
Effective Radiated Power (ERP) of the SFOL pulse with the nominal conditions of DME/N operation [24].

With the nominal DME/N operational conditions of 1000 Watts transmission power, Figure 7 shows the ERP of the SFOL pulse within the window of ±0.25 MHz at the frequency ranges in Equation (1). In Figure 7, the averaged ERPs at the frequencies ±0.8 and ±2.0 MHz away from the nominal channel frequency were 22 dBm and −11.5 dBm, respectively. Additionally, the averaged ERP monotonically decreased as the frequencies deviated from the center frequency. Therefore, the SFOL pulse satisfied the DME pulse spectrum requirements with the nominal operational conditions of DME/N.

### 4.2. Ranging Accuracy Assessment of the Stretched-Front-Leg (SFOL) Pulse

**Figure 8:**
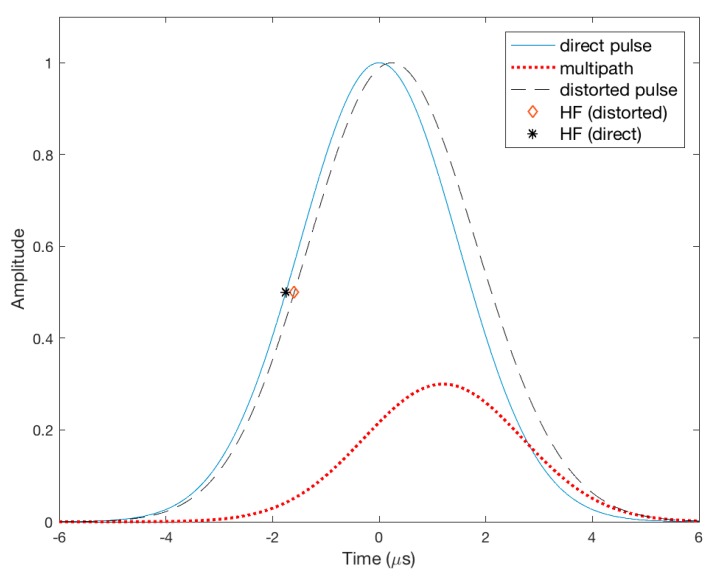
An example of the half amplitude point shifts of the Gaussian pulse due to multipath.

**Figure 9:**
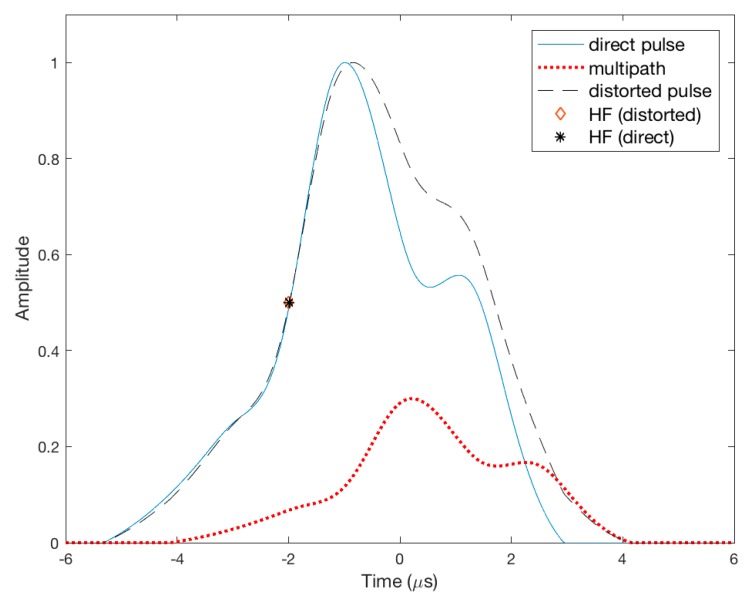
An example of the half amplitude point shifts of the SFOL pulse due to multipath.

The SFOL pulse was primarily designed to mitigate multipath effects through the proposed GA process. As an example of the effective multipath suppression capability of the SFOL pulse, Figure 8 and Figure 9 show the half amplitude point shifts (HAPS) due to multipath for the Gaussian and SFOL pulses. The time delay of the injected multipath was 1.2 μs and its phase difference with respect to the direct pulse was 0 degrees. Furthermore, the peak amplitude of the multipath was set to 30% of the direct pulse. In this multipath condition, the HAPS of the Gaussian pulse was 15.8 μs, which translated to a ranging error of 47.6 m. On the other hand, the HAPS of the SFOL pulse was −0.01 μs, which induced a −3.41 m ranging error. The reason that the SFOL pulse induced a small HAPS was that the rising edge of the SFOL pulse was barely distorted due to multipath. Rather, the multipath significantly distorted the falling edge of the SFOL pulse as shown in Figure 9, which did not impact the half amplitude point determination.

**Figure 10:**
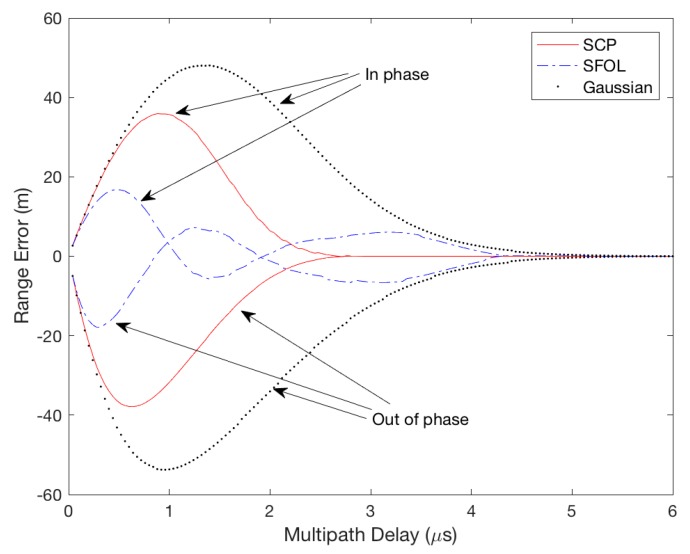
Range error envelopes due to in-phase and out-of-phase multipath for SCP, SFOL, and Gaussian pulses.

Figure 10 shows the envelop of the multipath-induced error for the Gaussian, SCP, and SFOL pulses in our simulations. The peak amplitude of the simulated multipath was again set to 30% of the direct pulse to represent a significant multipath environment [38]. The generated multipath had time delays varying from 0 to 6 μs in 1 ns time steps and had phase differences of 0 and 180 degrees with respect to the direct pulse. The multipath-induced ranging errors (RMS) of the envelope are summarized in Table 2. The SFOL pulse caused multipath-induced range errors of 77.3% and 60.4% less than the Gaussian and SCP pulses, respectively.

## 5. Discussion of Sensitivity and Distortion Sources

**Figure 11:**
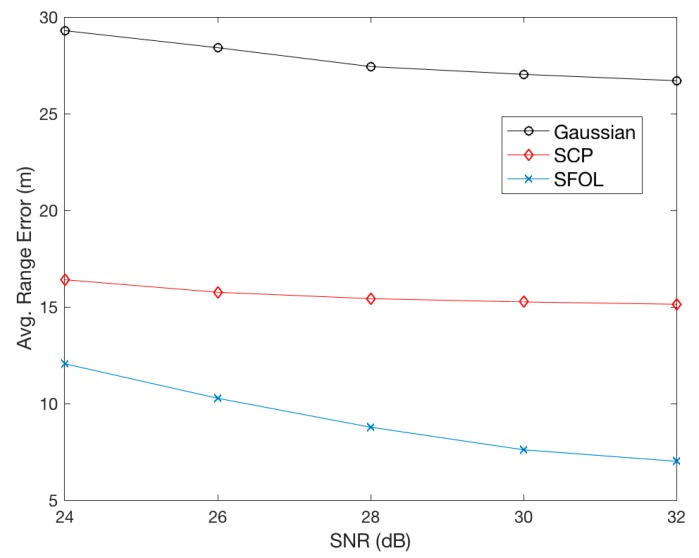
Averaged Root-Mean-Square (RMS) range errors in 1000 cases of noise injection with five levels of Signal to Noise Ratio (SNR).

In the previous section, the multipath mitigation performance was assessed using perfect pulse shapes without noise. In practice, the pulses will always be distorted due to additive noise caused by various sources such as receiver thermal noise. Therefore, it is necessary to see if the SFOL pulse would still retain the same level of advantage over the Gaussian and SCP pulses in a noisy environment. For this purpose, Gaussian white noise with a Signal-to-Noise Ratio (SNR) of 24 to 32 dB in 2 dB steps was generated and added to the direct and multipath signals. Figure 11 shows the resultant averaged range errors from 1000 cases of random noise simulation. At the SNR of 32 dB, the multipath mitigation performance of the SFOL pulse was close to that of the ideal pulse shape in the previous section. As the SNR decreased, the averaged range errors of the SFOL pulse distinctly degraded while those of the SCP pulse remained nearly constant. In fact, the SCP pulse was very insensitive to noise as its rising edge slope was consistently steep. However, the SFOL pulse started with a slow gradual slope that made the SFOL pulse suffer more from noise than the SCP and Gaussian pulse, although the slow gradual slope was effective in multipath mitigation. However, as seen in Figure 11, the SFOL pulse still reduced multipath-induced range errors by 56.9% and 36.0% less than the Gaussian and SCP pulses, respectively, even at the SNR of 24 dB.

Note that there are other possible sources of distortion when using the proposed SFOL pulse in practice. Particularly, the front-end filter in a DME receiver may require much wider bandwidth than that of the Gaussian pulse based DME equipment as the spectrum power of the SFOL pulse is higher. In addition, the hardware and software related to a power amplifier may require significant updates to transmit a reasonable SFOL pulse. However, these two problems may only be an issue for legacy DMEs. Modern DMEs are most likely advanced enough to properly process the SFOL pulse without much change [26]. In particular, the addition of a predistortion filter that cancels out the distortion introduced by a power amplifier would simplify the adaptation of the SFOL pulse [39].

For hard optimization problems with difficult characteristics, efficient solutions are not usually available, but GA can be excellent methods to apply [40]. Although the global optimality of GA solutions is not mathematically guaranteed likewise other heuristics-based algorithms, GA can provide reasonably good solutions in practice. There are several strategies in GA to overcome a local optimum. GA processes start from various initial populations, and the randomness allowed in GA—including mutation—also helps to prevent a local optimum. In this work, the initial population range was controlled by $\sigma_{R}$, $\sigma_{F}$, and ρ in Equations (5) and (6). During the implementation of the proposed GA process, $\sigma_{R}$ and $\sigma_{F}$ changed from 0.77 × 10^−6^ to 1.77 × 10^−6^ and from 4.8 × 10^−6^ to 5.5 × 10^−6^, respectively, in small steps. ρ changed from 0.3 to 0.8. It is mathematically uncertain that the SFOL is a globally optimal solution, but the SFOL pulse provides a significantly higher ranging accuracy than the SCP and Gaussian pulses, which are the prior state-of-the-art designs.

## 6. Conclusions

This study introduced an alternative DME pulse, called a SFOL pulse, that could significantly mitigate multipath effects. The SFOL pulse was developed by applying Genetic Algorithms and its design methodology was presented in detail. The SFOL pulse complied with the current DME pulse shape and spectrum requirements. With an ideal pulse shape, the SFOL pulse resulted in 77.3% and 60.4% less multipath-induced range errors than the Gaussian and SCP pulses. The sensitivity analysis that considered noise showed that the performance of the SFOL pulse degraded and its advantage over the Gaussian and SCP pulses diminished as the SNR decreased. However, the SFOL pulse still induced 56.9% and 36.0% less ranging errors than the Gaussian and SCP pulses due to multipath at a noisy environment of 24 dB SNR.

## Figures and Tables

**Table 1:** Distance Measuring Equipment (Normal) ground transponder pulse shape requirements [31].

Pulse Shape Parameters	Range
Rise Time	2.5 (+0.5, −1.0) μs
Pulse Top	No instantaneous fall below a value which is 95% of the maximum voltage amplitude of the pulse
Pulse Duration (width)	3.5 (±0.5) μs
Fall Time	2.5 (±0.5) μs

**Table 2:** Ranging error due to in-phase and out-of-phase multipath for Gaussian, SCP, and SFOL pulses.

Pulse	RMS (m)	Maximum (in Phase, m)	Maximum (out of Phase, m)
Gaussian	26.1	48.0	−53.75
SCP	14.9	35.9	−37.9
SFOL	5.9	16.8	−17.9
